# Supplementary material for: Preoperative Neutrophil-Lymphocyte Ratio for predicting surgery-related acute kidney injury in non-cardiac surgery patients under general anaesthesia: A retrospective cohort study
Source: PLoS One. 2022 Jul 29;17(7):e0270066. doi: 10.1371/journal.pone.0270066 (PMC9337669; doi:10.1371/journal.pone.0270066)
Supplement: Attachment — Submitted filename: Response to Reviewers.docx [file pone.0270066.s002.docx]

Re. MS # PONE-D-21-35925

Dear Giuseppe Filiberto Serraino:
We are submitting a revision of our manuscript entitled “Preoperative Neutrophil-Lymphocyte Ratio for Predicting Surgery-Related Acute Kidney Injury in Non-cardiac Surgery Patients under General Anaesthesia: A retrospective cohort study” for possible publication in PLOS ONE. We thank the Editor and Reviewers for their thoughtful and constructive comments on the initial submission. In response to the comments, we have addressed their critiques point-by-point as detailed below.

Reviewer #1: Thanks to the authors for their efforts and for touching on an important issue in the practice of nephrology.

Although it is a retrospective study, it consolidates the increasing evidence of the utility of NLR in predicting AKI.
**A:** We thank the Reviewer for the comments and have addressed the Reviewer’s other concerns below.

Q1: Can the authors elaborate more on the pre-op creatinine values?

**A:** The baseline serum creatinine level was calculated using the lowest level at preoperative day 7 and detected by the L-type creatinine M (Fujifilm Wako Pure Chemical Corporation, Japan).

Q2. What do you think is the reason for the low specificity of 51.9%?

**A:** The cut-off value of NLR differs in different patients (PMID: 33834972), the specificity and sensitivity also varies. The possible reason of the low specificity in our study might be the cut-off value and the type of surgery.

Q3. It would be better to mention data about the utility in Cardiovascular procedures, like CABG or repair of AA or in the HBP surgeries.

**A:** Thanks for your suggestion! We added the results of the NLR in cardiovascular surgery in our manuscript (Page 10, Main Document - marked copy).

Q4. Authors should mention data, if any, about the use of NLR in the post-op setting to compare with their results.

**A:** Thanks for your advice! We mentioned the study of postoperative NLR in the revised manuscript (Page 10, Main Document - marked copy).

Q5. It would be better to refer to the following paper, Chinese; with Meta-Analysis data: Lu Z, Wang L, Jia L, Wei F, Jiang A. [A Meta-analysis of the predictive effect of neutrophil-lymphocyte ratio on acute kidney injury]. Zhonghua Wei Zhong Bing Ji Jiu Yi Xue. 2021 Mar;33(3):311-317. Chinese. DOI: 10.3760/CMA.j.cn121430-20201215-00755. PMID: 33834972.

**A:** Thanks for your suggestion! We added this reference in our manuscript (Page 10, Main Document - marked copy).

Q6. The meta-analysis mentioned above has provided data about ethnic differences in NLR values (Asia vs. Eurasia). The authors can compare their cohort with the other groups.

**A:** The meta-analysis showed that increased NLR had predictive value for the occurrence of AKI in patients from Southeast Asia (MD = 4.04, 95%CI was 1.09-6.99, P = 0.007) and Eurasia (MD = 2.51, 95%CI was 1.12-3.90, P = 0.0004). The patients in our study were all from China, and the results were similar with the meta-analysis.

Reviewer #2: The neutrophil to lymphocyte ratio in various diseases had been discussed in detail in the past years. However, this important work is the first to suggest that it's preoperative value may predict postoperative AKI in non-cardiac surgery.

**A:** We thank the Reviewer for the comments and have addressed the Reviewer’s other concerns below.

There are two papers that may increase the value of "Discussion" if mentioned.

The work of Yu Y, et al. described similar results, but the NLR was measured postoperatively in the first 24 hours. They studied a much smaller number of patients.

Yu Y, Cui WH, Cheng C, Lu Y, Zhang Q, Han RQ. Association between neutrophil-to-lymphocyte ratio and major postoperative complications after carotid endarterectomy: A retrospective cohort study. World J Clin Cases. 2021 Dec 16;9(35):10816-10827. doi: 10.12998/wjcc.v9.i35.10816. PMID: 35047593; PMCID: PMC8678856.

The paper of Guangqing Z, et al. found the same result but in cardiac surgery. This recent paper probably was not published before the preparation of this paper.

Guangqing Z, Liwei C, Fei L, Jianshe Z, Guang Z, Yan Z, Jianjun C, Ming T, Hao C, Wei L. Predictive value of neutrophil to lymphocyte ratio on acute kidney injury after on-pump coronary artery bypass: a retrospective, single-center study. Gen Thorac Cardiovasc Surg. 2022 Feb 1. doi: 10.1007/s11748-022-01772-z. Epub ahead of print. PMID: 35103920.

**A:** Thanks for your suggestion! We added these two papers in our Discussion section (Page 10, Main Document - marked copy).

A few spelling mistakes:

line 94: Diabetes;

line 136: Low;

line 142: Preoperative do not need capital letter.

**A:** We are sorry about the mistake! We corrected these spelling mistakes in the revised version (Page 6, 8, Main Document - marked copy)

Once again, we thank the Editor and Reviewers for critically reviewing this study and for your constructive comments. We also make minor changes to our funding information in our revised manuscript. We look forward to hearing from you in the near future.

Sincerely,

Dan Li

Department of Anesthesiology

The Third Xiangya Hospital, Central South University

138 Tongzipo Road, Changsha,

Hunan，410013, China
